# Supplementary material for: cAmbly: A non-toxic cell-penetrating peptide derived from Amblyomin-X with targeted delivery to mitochondrial and cytoplasmic proteins
Source: PLoS One. 2025 Mar 12;20(3):e0318119. doi: 10.1371/journal.pone.0318119 (PMC11902296; doi:10.1371/journal.pone.0318119)
Supplement: Attachment — Submitted filename: Revision.docx [file pone.0318119.s001.docx]

Major points:

1. The abstract provides a good overview of the study however it could benefit from improved clarity and emphasis on the significance of the findings. The abstract should clearly state the significance of the finding for example, colocalizing with mitochondria, with preference for targeting mitochondrial proteins. The importance could be emphasized more. Also,the broader implications of the findings which is use of C-terminal of cAmbly for coupling molecules inside cells should be presented with more clarity and its potential impact on the field.
2. Line 64-66: Clarify which native and modified molecules were synthesized (e.g., specify the precise sequences of each peptide and there extensions in a figure. It may be helpful to provide a brief description of the properties of the native cAmbly sequence.
3. The MTT assay section is clear but it should be supplemented with how cell viability was calculated based on spectrophotometric readings and any controls used in the experiment. Also, consider specifying the number of replicates conducted for each assay to ensure the results' reliability and reproducibility.
4. Consider specifying the solvent control concentration Line 146: Provide more detail on how cell viability was calculated and how normalization was performed. Explain the significance of the lack of cytotoxicity for cAmbly. Line 149-151: The increase in activity of reducing enzymes in cells treated with cAmbly is an interesting finding. However, the implications of this result should be explained further. For instance, why is this increase significant? Does it suggest a potential benefit of cAmbly treatment? The reducing enzymes could be explained more to specify which reducing enzymes and their relevance. Provide more context for the results, particularly in relation to existing literature or expectations. How do these results compare to similar studies or to known effects of other CPPs?
5. Specify whether the UV light exposure is meant to crosslink the peptides with their targets in the cells or on the cell surface. provide information on how long the UV light exposure lasted. Also, consider adding more details about the fixation process (duration and temperature). Mention the concentrations of Hoechst, Phalloidin conjugated to Alexa Fluor 647, and streptavidin conjugated to Alexa Fluor 488 used for labeling. Consider specifying the number of replicates.
6. Mention the concentration of cAmbly-FITC and Mitotracker Deep Red used in the Mitochondrial Colocalization assay. Also, specify the duration of treatment. Provide details on the confocal microscopy imaging conditions, such as settings used for each fluorophore, and how the images were analyzed.
7. Specify the concentrations of native and modified cAmbly peptides used in the assay. Include a description of the controls used in the chemoproteomic assay, such as untreated cells or cells treated with non-modified peptides. Specify the concentration or volume of trypsin used for protein digestion. Provide more information about TMT-tagging, such as the specific reagents or kits used. Clarify the desalinization process using SDB-XC stage-tips (e.g., duration, flow rate). Include more details about the qExactivePlus mass spectrometer settings, including any specific parameters used for data acquisition and analysis. Clarify the methods used for protein quantification, such as specific algorithms or statistical models.
8. It was nowhere mentioned why mitochondria are of interest in this study. Consider stating the reason for focusing on mitochondrial colocalization specifically and why it is important. The lines 193-194 is not clear, present and explain the interpreted results more clearly. Provide more context for the results, such as how these findings compare to existing literature and discuss the implications of the colocalization results for potential therapeutic applications.
9. Chemoproteomic for cAmbly target discovery, explain briefly the mass spectrometry-based proteomics approach used, clarify the methods used for protein quantification. Explain the competitive assay in more detail. For example, describe how the concentrations of non-modified peptides were increased and what the expected outcomes were.

Minor points:

Line 14-15: "The delivery of drugs is a challenge when thinking about new molecules development" could be rephrased for clarity.

Line 16, “outstand this due its capacity" should be revised for clarity. Consider rephrasing to for example: "stand out due to their ability."

Line 25: "Delivery of some types of drugs are not always an easy task" could be improved for clarity. Consider rephrasing for example to "Delivering some types of drugs can be challenging."

Line 37: "small peptides, non-toxic, with up to 40 amino acids" could be improved for clarity. Consider rephrasing for example "small, non-toxic peptides consisting of up to 40 amino acids."

Line 57: "the Kunitz domain could not enter and kill the tumor cell" could gain from being more explicit about the interaction or mechanism involved.

Line 68-70: Explain the rationale for modifying the N-terminal or C-terminal of the peptide and how the design was expected to affect function.

Line 65: Rephrase "one native and three modified molecules of cAmbly peptide" to "one native cAmbly peptide and three modified variants."

Line 69: Rephrase "for purification and labeling purposes" to "for purification and labeling."

Line 73-77: The cell culture section provides the basic conditions for cell growth, also specify the number of cells seeded per well and any other relevant culture conditions.

Line 100: Mention the concentration of cAmbly-FITC and Mitotracker Deep Red used in the assay. Also, specify the duration of treatment.

Line 106: "At 100% confluency" could benefit from specifying the number of cells or density to provide more context on cell seeding.

Line 107: Specify the concentrations of native and modified cAmbly peptides used in the assay.

Line 115: Consider rephrasing "qExactivePlus mass spectrometer coupled to a nanoEasy 1200 chromatograph" for clarity.
