# Supplementary material for: cAmbly: A non-toxic cell-penetrating peptide derived from Amblyomin-X with targeted delivery to mitochondrial and cytoplasmic proteins
Source: PLoS One. 2025 Mar 12;20(3):e0318119. doi: 10.1371/journal.pone.0318119 (PMC11902296; doi:10.1371/journal.pone.0318119)
Supplement: Attachment — Submitted filename: Response_to_Reviewers_auresp_4.docx [file pone.0318119.s004.docx]

August 23^th^, 2024

PLOS ONE

Dear Editor:

We wish to re-submit an original research article for publication in PLOS ONE, titled “cAmbly, a non-toxic cell-penetrating peptide (CPP) derived from Amblyomin-X, targeting mitochondrial and cytoplasmic proteins.” The paper was co-authored by Marcus V. Buri, Graciana Yokota Garavelli, Hugo Vigerelli, Marcelo M. de Souza, Aline R. M. Lobba, Sonja Ghidelli-Disse, and Ana M. Chudzinski-Tavassi

A recent study showed that the C-terminal portion of Amblyomin-X, an antitumoral protein, was responsible for the internalization of the protein into Tumoral cells. This report presents the potential cell-penetrating properties of the C-terminal of Amblyomin-X, here called cAmbly. We showed that cAmbly was able to internalize T98G cells from 30 minutes of incubation, colocalizing with mitochondria, with a preference for targeting mitochondrial proteins. We also showed that the C-terminal of cAmbly is preferred for coupling molecules to be delivered inside the cells. We showed in this article that even though it is not cytotoxic, targeting mitochondria and mitochondrial proteins cAmbly can deliver drugs to mitochondria, triggering its mechanism of action, for instance, apoptosis.

We would like to answer the major points mentioned by the reviewers:

**1.** The abstract provides a good overview of the study however it could benefit from improved clarity and emphasis on the significance of the findings. The abstract should clearly state the significance of the finding for example, colocalizing with mitochondria, with preference for targeting mitochondrial proteins. The importance could be emphasized more. Also,the broader implications of the findings which is use of C-terminal of cAmbly for coupling molecules inside cells should be presented with more clarity and its potential impact on the field.

**Ans.** We agree with the comment, and we added the potential of cAmbly as a cargo delivery for the mitochondria in the abstract and conclusion

**2.** Line 64-66: Clarify which native and modified molecules were synthesized (e.g., specify the precise sequences of each peptide and there extensions in a figure. It may be helpful to provide a brief description of the properties of the native cAmbly sequence.

**Ans.** We added a better explanation of peptide synthesis and an image about it

**3.** The MTT assay section is clear but it should be supplemented with how cell viability was calculated based on spectrophotometric readings and any controls used in the experiment. Also, consider specifying the number of replicates conducted for each assay to ensure the results' reliability and reproducibility.

**Ans.** We better explained the MTT method

**4.** Consider specifying the solvent control concentration Line 146: Provide more detail on how cell viability was calculated and how normalization was performed. Explain the significance of the lack of cytotoxicity for cAmbly. Line 149-151: The increase in activity of reducing enzymes in cells treated with cAmbly is an interesting finding. However, the implications of this result should be explained further. For instance, why is this increase significant? Does it suggest a potential benefit of cAmbly treatment? The reducing enzymes could be explained more to specify which reducing enzymes and their relevance. Provide more context for the results, particularly in relation to existing literature or expectations. How do these results compare to similar studies or to known effects of other CPPs?

**Ans.** We explained in more detail about the MTT experiment. The cAmbly did not affect cell viability, and the slight increase in formazan formation led us to further investigate the effect on mitochondria

**5**. Specify whether the UV light exposure is meant to crosslink the peptides with their targets in the cells or on the cell surface. provide information on how long the UV light exposure lasted. Also, consider adding more details about the fixation process (duration and temperature). Mention the concentrations of Hoechst, Phalloidin conjugated to Alexa Fluor 647, and streptavidin conjugated to Alexa Fluor 488 used for labeling. Consider specifying the number of replicates.

**Ans.** The crosslink with 5J/cm2 of UV light is sufficient for covalently binding the peptide with its target through diazirine reaction, wherever the peptide is, inside the cell or at the surface, mimicking interaction with the natural target of the peptide

**6.** Mention the concentration of cAmbly-FITC and Mitotracker Deep Red used in the Mitochondrial Colocalization assay. Also, specify the duration of treatment. Provide details on the confocal microscopy imaging conditions, such as settings used for each fluorophore and how the images were analyzed.

**Ans.** All the concentrations were added to the methods section

**7.** Specify the concentrations of native and modified cAmbly peptides used in the assay. Include a description of the controls used in the chemoproteomic assay, such as untreated cells or cells treated with non-modified peptides. Specify the concentration or volume of trypsin used for protein digestion. Provide more information about TMT-tagging, such as the specific reagents or kits used. Clarify the desalination process using SDB-XC stage-tips (e.g., duration, flow rate). Include more details about the qExactivePlus mass spectrometer settings, including any specific parameters used for data acquisition and analysis. Clarify the methods used for protein quantification, such as specific algorithms or statistical models.

**Ans.** We better explained the chemoproteomic assay and the competition analysis to include the requested explanations

**8.** It was nowhere mentioned why mitochondria are of interest in this study. Consider stating the reason for focusing on mitochondrial colocalization specifically and why it is important. The lines 193-194 is not clear, present and explain the interpreted results more clearly. Provide more context for the results, such as how these findings compare to existing literature and discuss the implications of the colocalization results for potential therapeutic applications.

We would like to thank you for this review and added some points about the importance of targeting mitochondria and proteins for cAmbly peptide

**9.** Chemoproteomic for cAmbly target discovery, explain briefly the mass spectrometry-based proteomics approach used, clarify the methods used for protein quantification. Explain the competitive assay in more detail. For example, describe how the concentrations of non-modified peptides were increased and what the expected outcomes were.

**Ans.** We better explained the chemoproteomic assay and the competition analysis to include the requested explanations.

**10.** Thank you for updating your data availability statement. You note that your data are available within the Supporting Information files, but no such files have been included with your submission. At this time we ask that you please upload your minimal data set as a Supporting Information file, or to a public repository such as Figshare or Dryad.

Please also ensure that when you upload your file you include separate captions for your supplementary files at the end of your manuscript.

As soon as you confirm the location of the data underlying your findings, we will be able to proceed with the review of your submission.

**Ans.** We are uploading the raw files of chemoproteomic assay with the caption at the manuscript

**12.** When completing the data availability statement of the submission form, you indicated that you will make your data available on acceptance. We strongly recommend all authors decide on a data sharing plan before acceptance, as the process can be lengthy and hold up publication timelines. Please note that, though access restrictions are acceptable now, your entire data will need to be made freely accessible if your manuscript is accepted for publication. This policy applies to all data except where public deposition would breach compliance with the protocol approved by your research ethics board. If you are unable to adhere to our open data policy, please kindly revise your statement to explain your reasoning and we will seek the editor's input on an exemption. Please be assured that, once you have provided your new statement, the assessment of your exemption will not hold up the peer review process.

**Ans.** All data can be shared after publication without restrictions

The funders had no role in study design, data collection and analysis, decision to publish, or preparation of the manuscript.

**13**. Please ensure that you include a title page within your main document. We do appreciate that you have a title page document uploaded as a separate file, however, as per our author guidelines (http://journals.plos.org/plosone/s/submission-guidelines#loc-title-page) we do require this to be part of the manuscript file itself and not uploaded separately.

Could you therefore please include the title page into the beginning of your manuscript file itself, listing all authors and affiliations.

**Ans.** The title and authors was added along with the manuscript

**14**. Thank you for stating the following financial disclosure:

[This research was funded by “Grant 2015/50040-4, São Paulo Research Foundation and GlaxoSmithKline”, “Grant 2020/13139-0, São Paulo Research Foundation and GlaxoSmithKline”, and Grant number 2013/07467-1 (FAPESP-CETICs). A.M.C.-T. is a recipient of CNPq-PQ grant number 303197/2017-0 and Fundação Butantan-PQ grants. R.N.G. is a recipient of FAPESP grant number 2018/20469-7. A.M.A is a recipient of FAPESP grant number 2018/10937-3.].

Please state what role the funders took in the study. If the funders had no role, please state: ""The funders had no role in study design, data collection and analysis, decision to publish, or preparation of the manuscript.""

If this statement is not correct you must amend it as needed.

Please include this amended Role of Funder statement in your cover letter; we will change the online submission form on your behalf.

**Ans.** We added the statement "The funders had no role in study design, data collection and analysis, decision to publish, or preparation of the manuscript."

**Other comments:** None of our images have copyrights since every art and data were created or generated by ourselves

Further, we would also like to thank the reviewers whose contributions made the article better and more accessible to readers. We want to publish this article in this journal because PlosOne has a broad reach. We believe that the evolution of CPPs can help the development and delivery of drugs against various diseases, being cAmbly a potential candidate for this kind of development, specifically if it is intended to target mitochondria, helping the scientific community solve several health problems.

This manuscript has not been published or presented elsewhere in part or in entirety and is not under consideration by another journal. The appropriate ethics review board approved the study design. We have read and understood your journal’s policies, and we believe that neither the manuscript nor the study violates any of these. There are no conflicts of interest to declare.

**ACADEMIC EDITOR:** Authors have answered most of the reviweres comments. However, quality of figures are still need significant improvement in terms of resolution. It's hard to read figure axis in some places.

**Ans.** We concur with the suggestions of the Academic Editor and have made adjustments to the figures to enhance their resolution. Figures 4 and 5 have been replaced with higher-resolution images, and Figure 7 has been redrawn to improve the legibility of the axes.

Thank you for your consideration. I look forward to hearing from you.

Sincerely,

Ana Marisa Chudzinski-Tavassi

Instituto Butantan, Sao Paulo, SP, Brazil

Phone: +55 11 2627-9555 / email: ana.chudzinski@butantan.gov.br
